# Phthalate Exposure Changes the Metabolic Profile of Cardiac Muscle Cells

**DOI:** 10.1289/ehp.1205056

**Published:** 2012-06-06

**Authors:** Nikki Gillum Posnack, Luther M. Swift, Matthew W. Kay, Norman H. Lee, Narine Sarvazyan

**Affiliations:** 1 Department of Pharmacology and Physiology, and; 2 Department of Electrical and Computer Engineering, The George Washington University, Washington, DC, USA

**Keywords:** di(2-ethylhexyl)phthalate, cardiomyocyte, fatty acid metabolism, microarray, plasticizer

## Abstract

Background: Phthalates are common plasticizers present in medical-grade plastics and other everyday products. They can also act as endocrine-disrupting chemicals and have been linked to the rise in metabolic disorders. However, the effect of phthalates on cardiac metabolism remains largely unknown.

Objectives: We examined the effect of di(2-ethylhexyl)phthalate (DEHP) on the metabolic profile of cardiomyocytes because alterations in metabolic processes can lead to cell dysfunction.

Methods: Neonatal rat cardiomyocytes were treated with DEHP at a concentration and duration comparable to clinical exposure (50–100 μg/mL, 72 hr). We assessed the effect of DEHP on gene expression using microarray analysis. Physiological responses were examined via fatty acid utilization, oxygen consumption, mitochondrial mass, and Western blot analysis.

Results: Exposure to DEHP led to up-regulation of genes associated with fatty acid transport, esterification, mitochondrial import, and β-oxidation. The functional outcome was an increase in myocyte fatty acid–substrate utilization, oxygen consumption, mitochondrial mass, PPARα (peroxisome proliferator-activated receptor α) protein expression, and extracellular acidosis. Treatment with a PPARα agonist (Wy-14643) only partially mimicked the effects observed in DEHP-treated cells.

Conclusions: Data suggest that DEHP exposure results in metabolic remodeling of cardiomyocytes, whereby cardiac cells increase their dependence on fatty acids for energy production. This fuel switch may be regulated at both the gene expression and posttranscription levels. Our findings have important clinical implications because chronic dependence on fatty acids is associated with an accumulation in lipid intermediates, lactate, protons, and reactive oxygen species. This dependence can sensitize the heart to ischemic injury and ventricular dysfunction.

An estimated 34% of U.S. adults have metabolic syndrome, which can be a precursor to diabetes, obesity, heart disease, stroke, and cancer (Ervin 2009). Although the role of lifestyle choices in these disorders cannot be ignored, the dramatic rise in metabolic disease suggests that environmental pollutants may also play a role. Endocrine-disrupting chemicals can interfere with endocrine function, resulting in adverse developmental, reproductive, and metabolic effects. Human exposure to endocrine disruptors has been linked to metabolic disturbances (reviewed by Casals-Casas and Desvergne 2011). Endocrine disruptors are known to interact with nuclear receptors, which modulate downstream gene expression by interfering with receptor function and/or transcription factor activity.

Di(2-ethylhexyl)phthalate (DEHP) is a commonly used plasticizer that imparts flexibility to polyvinyl chloride (PVC) products. It has been identified as an endocrine-disrupting chemical (Casals-Casas and Desvergne 2011). Human exposure to DEHP occurs through contact with food packaging, toys, and personal care and medical products. The presence of DEHP in medical products is of particular concern because exposure to DEHP increases dramatically in patients undergoing multiple medical interventions, such as cardiopulmonary bypass, hemodialysis, transfusions, and other procedures that require extensive use of tubing in intensive care units. This is because DEHP is not covalently bound to the PVC polymer and is highly hydrophobic, allowing it to leach from plastics when in contact with blood and other lipophilic fluids [Food and Drug Administration (FDA) 2002]. Several animal studies have reported toxic effects of DEHP (reviewed by Carlson 2010), raising concerns about phthalate leaching and human health. Exposure to DEHP results in broad phthalate distribution throughout the body, including the heart (Hillman et al. 1975). Our recent studies revealed that DEHP adversely affects the synchronicity of a cardiac cell network by disrupting connexin-43, the main component of cardiac gap junctions (Gillum et al. 2009). This effect, which may be attributed to modifications in tubulin and kinesin expression, as well as other gene expression modifications, suggested an arrhythmogenic effect of phthalates *in vitro* (Posnack et al. 2011). We sought to expand these studies to examine DEHP’s effect on the metabolic profile of cardiomyocytes because metabolic alterations can point to additional dysfunctions.

To meet high energy demands, cardiac muscle is able to metabolize multiple substrates. Fatty acids (FAs) are the preferred substrate: At least 60% of ATP is generated from FA oxidation (FAO) (reviewed by Lopaschuk et al. 2010). The remaining energy demand is provided by glucose, lactate, and ketone metabolism. Several studies have addressed the effects of phthalates on metabolism (Casals-Casas et al. 2011; Desvergne et al. 2009; Stahlhut et al. 2007; Svensson et al. 2011). In contrast, the effect of DEHP on cardiac FA metabolism remains largely unknown (Itsuki-Yoneda et al. 2007; Reubsaet et al. 1990). We found that DEHP exposure results in an up-regulation of genes associated with FAO. DEHP-induced genetic modifications resulted in increased FA substrate utilization, increased mitochondrial mass, increased oxygen consumption, and extracellular acidosis. Data suggest that these modifications involve up-regulation of PPARα (peroxisome proliferator-activated receptor α) and its coactivator PGC-1α (peroxisome proliferator-activated receptor γ, coactivator 1α), as well as alternative pathways.

## Materials and Methods

All animals were treated humanely and with regard for alleviation of suffering.

*Experimental protocol.* Cardiomyocytes were isolated from mixed litters of 1-day-old Sprague-Dawley rats (approximately 25 rats/litter; Hilltop Lab Animals, Scottdale, PA) by an enzymatic digestion procedure (Arutunyan et al. 2001). Cells were treated with either dimethyl sulfoxide (DMSO; control), 50 μg/mL DEHP (128 μM), 3) 100 μg/mL DEHP (256 μM), or 50 μM Wy-14643 (PPARα agonist). Unless otherwise noted, cells were treated for 72 hr before conducting experiments. Cell toxicity was assessed using a membrane integrity assay (CytoTox-ONE; Promega, Madison WI), and the effects on cell proliferation were assessed by measuring lactate dehydrogenase (LDH) release.

*Microarrays and validation by quantitative real-time polymerase chain reaction (qRT-PCR*). Microarray experiments (Rat 1.0 ST array; Affymetrix, Santa Clara, CA) were performed using six coverslips of cardiomyocytes for each treatment group (control and 50 μg/mL DEHP) as previously described (Posnack et al. 2011). Hybridization data are available from the Gene Expression Omnibus (accession GSE21640) (Edgar et al. 2002). We identified differentially regulated genes by Student’s *t*-test, with a 1% false-discovery rate and an expression difference cut-off of 1.5. GeneSpring software (Agilent Technologies Inc., Santa Clara, CA) was used to identify Gene Ontology (GO) categories (*p* < 0.01), and a polyhierarchical graph specific for the metabolic process category was created and modified using AmiGO visualization (Carbon et al. 2009). TIGR Multiexperiment Viewer (MeV) software (version 4.7.4; Saeed et al. 2006) was used to visualize genes by GO category, using logarithm (base 2) values with median background correction. Ingenuity Pathway Analysis software (IPA; Ingenuity Systems Inc., Redwood City, CA) and PathVisio (van Iersel et al. 2008) was used to identify gene networks and canonical pathways. Microarray experiments were validated using qRT-PCR as previously described (Posnack et al. 2011). Quantitation and normalization of relative gene expression was accomplished using the comparative CT method (ΔΔCT); ΔΔCT values were converted to ratios by 2^–ΔΔCT^ averaged across replicates. Primer sequences for acetyl-CoA acyltransferase (*ACAA2*), long-chain acyl-CoA dehydrogenase (*ACADL*), acyl-CoA synthetase (*ACSL1*), very long chain acyl-CoA dehydrogenase (*ACADVL*), carnitine palmitoyltransferase 1B (*CPT1B*), enoyl-CoA hydratase (*ECH1*), hydroxyacyl-CoA dehydrogenase (*HADHA*), pyruvate dehydrogenase kinase (*PDK4*), *PGC-1*α, *PGC-1*β, *PPAR*α, glutamate dehydrogenase, and 18S ribosomal RNA are available in Supplemental Material, Table S1 (http://dx.doi.org/10.1289/ehp.1205056).

*FA utilization.* Seventy-two hours after treatment with DMSO (control) or 50 μg/mL DEHP, cell culture media was changed to a glucose-free media supplemented with 100 μM palmitic acid and 33 μM bovine serum albumin (BSA). An increased rate of FA utilization was detected as a decrease in media concentration over time. FA concentration was measured over 30 hr using a fluorescence-based assay (Free Fatty Acid Quantification Kit; BioVision, Milpitas, CA) in which FAs were converted to coenzyme A (CoA) derivatives for quantification.

*Immunocytochemistry.* Cardiomyocytes were fixed using a standard 4% paraformaldehyde procedure and stained with PPARα (1:500) and anti-rabbit Cy3 (cyanine dye; 1:1,000). Live cells were loaded with MitoTracker Red (0.1 μM; Life Technologies, Grand Island, NY) and imaged at 20× magnification, or loaded and then fixed for high magnification imaging (63×).

*Western blot.* Blots were prepared as previously described (Posnack et al. 2011). Blots were probed with PPARα (1:300) and GAPDH (1:3,500; loading control). Blots were incubated with anti-rabbit IRdye 800CW (1:5,000) and anti-mouse IRdye 680LT (1:10,000), both from LI-COR Biosciences (Lincoln, NE).

*Proton production.* We prepared standards by adjusting the pH of media samples containing phenol red. Absorbance spectra were acquired for the standards and the cell culture samples using a plate reader. The cell culture samples were then normalized against the standards to identify pH modifications.

*Oxygen consumption.* Cardiomyocytes were plated on an XF24 cell culture microplate (100,000 cells/well; Seahorse Bioscience, North Billerica, MA). Cells were treated for 72 hr with DMSO or 50 μg/mL DEHP. An XF24 Analyzer (Seahorse Biosciences) was then used to measure oxygen consumption in real time. Prior to XF measurements, cells were incubated for 1 hr at 37°C in unbuffered media supplemented with *a*) 100 μM palmitic acid with 33 μM BSA, or *b*) 25 mM glucose. To validate these results independently, cells were loaded with an oxygen-sensitive probe (MitoXpress; Axxora, San Diego, CA) in media supplemented with palmitic acid and BSA. Mineral oil was used to seal the samples and diminish the interference of ambient oxygen. A high rate of cell respiration depletes the sample oxygen concentration quickly, which is detected as an increase in probe signal.

*Statistical methods.* Student’s *t*-test was used to evaluate the significance of the differences in mean values between two treatment groups. Analysis of variance and Bonferroni’s multiple comparison test were used to evaluate values between three or more groups. Values are presented as mean ± SE, with *p* < 0.05 considered statistically significant.

## Results

*Gene expression profile of DEHP-treated cardiomyocytes.* We studied the effect of DEHP on neonatal rat cardiomyocytes at a dose (50–100 μg/mL) comparable to plasticizer presence in neonates undergoing clinical procedures, such as transfusion or extracorporeal membrane oxygenation (FDA 2002; Gillum et al. 2009). After 72 hr exposure, RNA was harvested from cardiomyocytes for DNA microarray experiments. At a 1.5-fold expression difference cut-off, a total of 814 mRNAs were differentially expressed. Of these genes, 334 were up-regulated and 480 were down-regulated by DEHP treatment.

**Figure 1:**
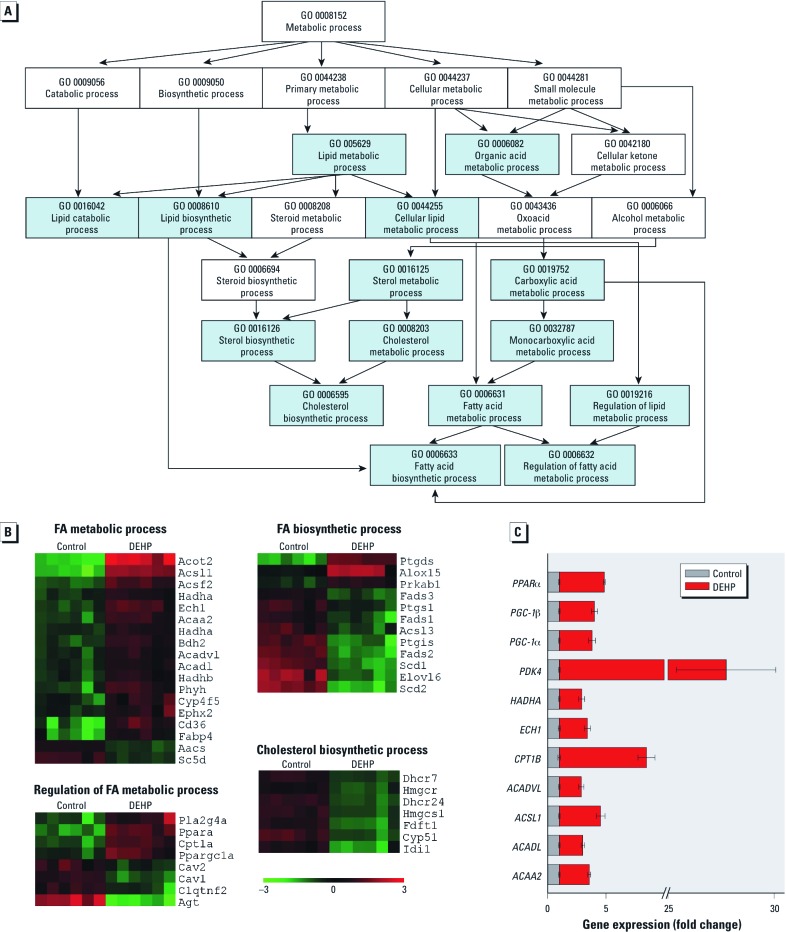
DEHP treatment (50 µg/mL) altered the expression of genes associated with metabolic and biosynthetic processes. (*A*) Polyhierarchical graph shows GO categories under the metabolic process ontology. Arrows show directionality of hierarchy. Blue boxes indicate GO categories that are significantly different (*p *< 0.01). (*B*) Heatmap images show differential gene expression in GO categories (*p *< 0.01). Abbreviations: Acaa2, *ACAA2*; Acadl, *ACADL*; Acsl1, *ACSL1*; Acadvl, *ACADVL*; Cpt1b, *CPT1B*; Ech1, *ECH1*; Hadha, *HADHA*; Pdk4, *PDK4*; Pgc1a, *PGC‑1*α; Pgc1b, *PGC‑1*β; Ppara, *PPAR*α. Each row represents an individual gene, and each column represents an independent experiment. Red indicates up‑regulated genes, and green indicates down‑regulated genes*.* (*C*) qRT‑PCR indicates genes associated with FA metabolism and regulation. Values are mean ± SE; changes were significantly different for all genes (*n *= 4).

Gene Ontology (GO) analysis revealed that differentially expressed genes were significantly overrepresented in 97 categories (*p* < 0.01). The lipid metabolic process GO category was the most significantly modified (*p* < 0.00001). Figure 1A shows a polyhierarchical graph specific for GO categories under the metabolic process ontology. We used MeV to identify genes significantly up- or down-regulated within four downstream GO categories (Figure 1B): cholesterol metabolism, FA metabolic process, regulation of FA metabolic process, and FA biosynthetic process. The latter two categories are subdivisions of FA metabolic process; genes that overlapped between categories are displayed only once in the heatmap images. In DEHP-treated samples, most genes associated with FA metabolic processes were up-regulated, whereas most genes associated with biosynthesis were down-regulated (Figure 1B).

*qRT-PCR validation.* The mRNA expression of 11 genes associated with FA metabolism and regulation were validated using qRT-PCR (Figure 1C). Genes associated with regulation of FA metabolism were up-regulated: *PPAR*α, *PGC-1*α, and *PGC-1*β. Genes encoding FA metabolic enzymes were also up-regulated: *ACSL1*, *ACAA2*, *ACADL*, *ACADVL*, and *HADHA*. *CPT1B*, which transports FAs into the mitochondria, was also up-regulated. We also detected an up-regulation in *ECH1*, an enzyme that functions in the auxiliary step of the β-oxidation pathway, and *PDK4*, a protein that regulates glucose metabolism.

**Figure 2:**
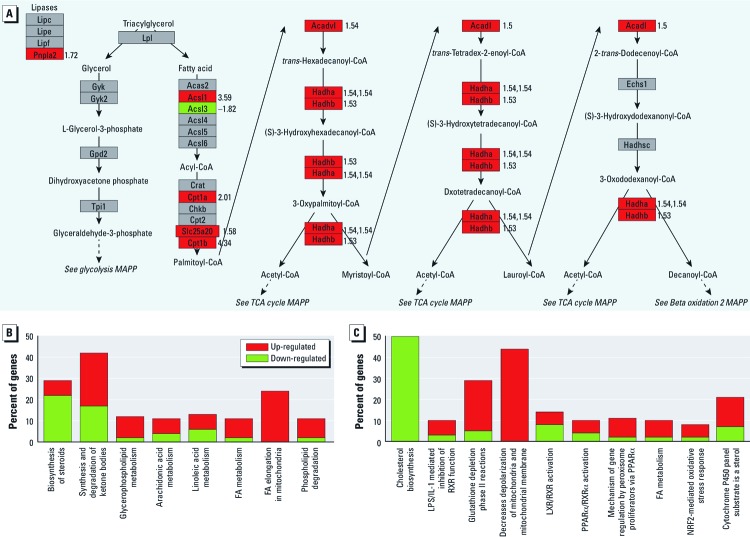
DEHP treatment (50 µg/mL) up‑regulated genes associated with FAO and modifies lipid canonical and detoxification pathways. (*A*) Mitochondrial FAO pathway overlaid with mRNA expression fold change values (numbers to right of each gene). Up‑regulation (red) is indicated by a positive value, and down‑regulation (green) is indicated by a negative value. (*B*) Modified canonical pathways associated with lipid metabolism, and (*C*) modified detoxification pathways.

*Gene network analysis.* We used IPA and PathVisio to identify biological pathways pertinent to lipid metabolism. A mitochondrial FAO pathway overlaid with mRNA expression fold-change values is shown in Figure 2A. Genes involved in multiple parts of the FAO pathway were up-regulated in DEHP-treated samples. These included triglyceride hydrolysis (*PNPLA2*), FA esterification (*ACSL1*), mitochondrial import (*CPT1A, CPT1B*, and *SLC25A20*) and β-oxidation (*ACADL, ACADVL, HADHA,* and *HADHB)*. Conversely, *ACSL3,* which encodes an acyl-CoA synthetase enzyme involved in FA biosynthesis, was down-regulated.

In addition to FA metabolism and FA elongation, alternative lipid canonical pathways were also altered, including those related to steroid, ketone, arachidonic acid, linoleic acid, and phospholipid metabolism and degradation (Figure 2B). IPA toxicity analysis also revealed functionally grouped gene sets that are altered in response to a xenobiotic exposure (DEHP). As expected, PPARα activation and FA metabolism genes were up-regulated (Figure 2C). In comparison, cholesterol biosynthesis was down-regulated. Gene sets associated with detoxification pathways, including glutathione depletion and cytochrome P450, were also up-regulated in DEHP samples. Toxicity gene sets associated with mitochondrial membrane depolarization and *NRF2* (NF-E2-related factor 2)-mediated oxidative stress response were also up-regulated. These are of interest because increased FAO is likely to enhance reactive oxygen species production, and NRF2 up-regulates antioxidant genes (Gao et al. 2007).

**Figure 3:**
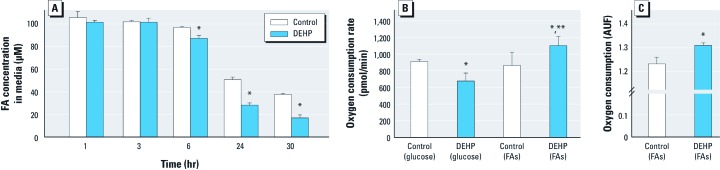
DEHP treatment (50 µg/mL) enhanced FA utilization and oxygen consumption. (*A*) Rate of FA utilization detected by a decrease in FA concentration over time (*n *= 3). (*B*) Oxygen consumption rates were maximal in DEHP samples supplemented with palmitic acid and minimal when supplemented with glucose. (*C*) Total oxygen consumption was increased in DEHP samples supplemented with FAs (*n *= 4); AUF, arbitrary units of fluorescence. Values are mean ± SE. *p < 0.05 compared with the corresponding control. **p < 0.05 compared with DEHP (glucose).

*Increased FAO and mitochondrial oxygen consumption.* To determine if gene expression changes resulted in a physiological response, we measured the rate of FA utilization during a 30-hr period by modifying the cell culture media to contain only FAs (glucose free). After 6 hr, DEHP-treated samples metabolized significantly more FAs, as indicated by a drop in FA concentration in cell media samples (Figure 3A). This effect became more pronounced at later time points.

Compared with glucose oxidation, FAO requires a greater amount of oxygen per molecule of ATP produced. Therefore, increased reliance on FA substrates for energy production should also increase oxygen consumption. To further investigate the effect of DEHP on FAO, we measured oxygen consumption rates in cardiomyocytes cultured with either 100 μM palmitic acid or 25 mM glucose using an extracellular flux analyzer. Oxygen consumption rates were increased in DEHP-treated samples supplemented with palmitic acid, and decreased in DEHP-treated samples supplemented with glucose (Figure 3B). This decrease in oxygen consumption may signify a lack of substrate flexibility in response to DEHP whereby glucose utilization is diminished. These data were independently validated by measuring total oxygen consumption using an oxygen-sensitive probe in the presence of 100 μM palmitic acid (Figure 3C).

**Figure 4:**
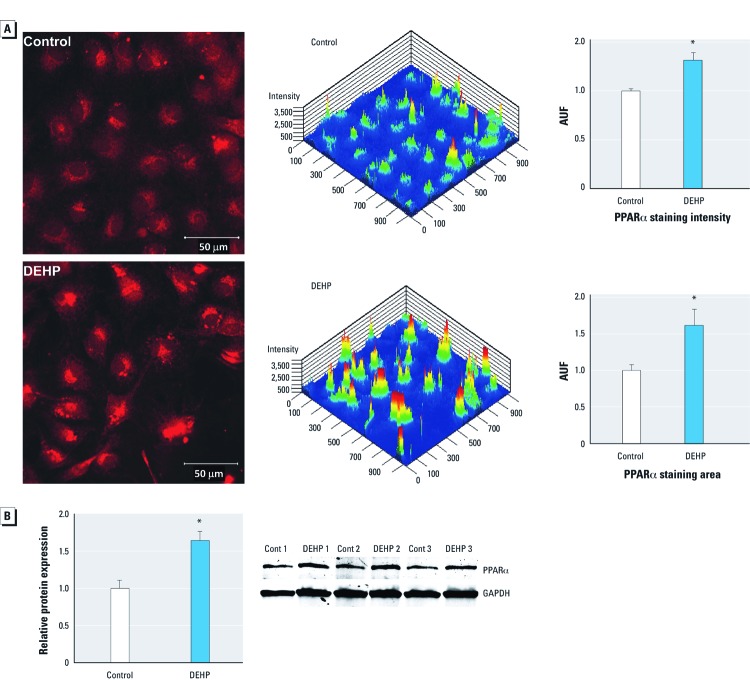
DEHP treatment (50 µg/mL) increased PPARα protein expression. (*A*) PPARα staining intensity and total area were increased in DEHP-treated samples as shown by immunostaining (red; left) and using rainbow color in a 2.5-dimensional plot (center; high intensity indicated by red and low intensity by blue); fluorescence values [arbitrary units of fluorescence (AUF); mean ± SE] for staining intensity and staining area are summarized in the graphs (right). (*B*) PPARα protein expression (mean ± SE) was increased in DEHP-treated samples normalized to GAPDH (*n *= 3). *p < 0.05 compared with the control.

*Increased PPAR*α *expression.* The PPARα nuclear receptor acts as a lipid sensor that modifies transcriptional responses to metabolic status. Activation (or inhibition) of PPARα via exogenous compounds can interfere with metabolic homeostasis. Phthalates are known PPARα agonists (Bility et al. 2004). PPARα protein expression was increased in DEHP-treated samples compared with the control, as shown by immunostaining (Figure 4A) and Western blot analysis (Figure 4B).

**Figure 5:**
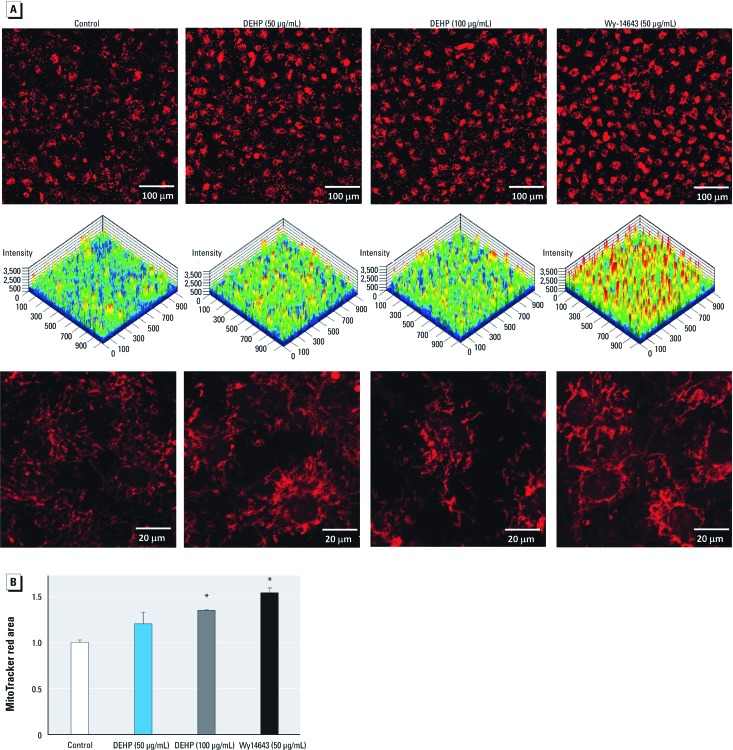
DEHP and Wy-14643 treatment increased mitochondria mass. (*A*) Live imaging of cells loaded with MitoTracker red showed increased staining in DEHP (100 μg/mL) or Wy-14643 samples (20×; top). Images shown in (*A*) represented in 2.5-dimensional plots, with staining intensity shown in rainbow color (high intensity indicated by red and low intensity by blue; center). Cells were fixed to eliminate contraction movement (63×; bottom). (*B*) The area with total mitochondrial staining (mean ± SE) was larger in samples treated with DEHP (100 μg/mL) or Wy-14643 (50 μM) (*n *= 3). *p < 0.05 compared with the control.

*Increased mitochondrial mass.* Activation of PPARα and its transcriptional coactivator protein, PGC-1α, can increase mitochondria biogenesis (Wu et al. 1999). Because both PPARα and PGC-1α are up-regulated in DEHP samples, we examined mitochondrial mass using MitoTracker. As a positive control, PPARα was activated directly via Wy-14643, a specific PPARα agonist (Ren et al. 2010). Assessment of mitochondrial structure showed an increase in mitochondrial mass, particularly in cells treated with a high concentration of DEHP (100 μg/mL) or Wy-14643 (Figure 5).

**Figure 6:**
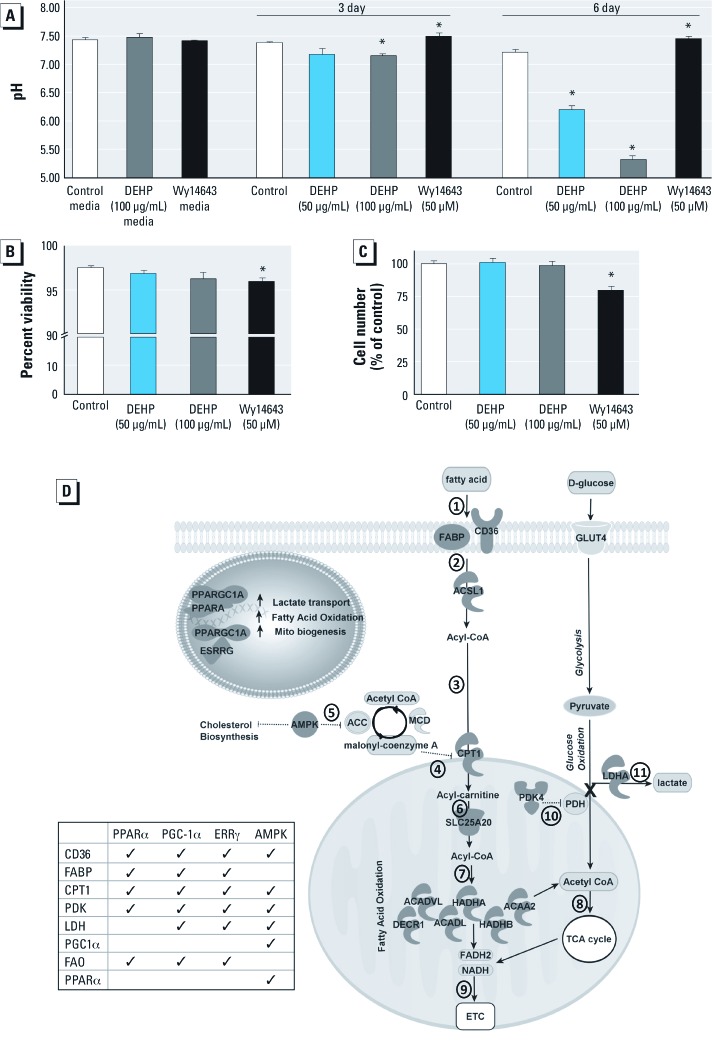
DEHP treatment resulted in proton accumulation (*A,B,C*), which is likely due to modifications in FA and glucose oxidation pathways (*D*). (*A*) pH measurements of media samples with chemical additives, either in the absence (media; left) or presence of cells (*n *= 3). (*B,C*) Decreased cell viability (2.5% of control; *B*) or reduction in cell number (20% of control; *C*) may explain pH changes in Wy-14643 samples but not DEHP samples; values are mean ± SE (*n *= 3 for each treatment)*. *(*D*) DEHP treatment increased the expression of multiple genes in the FAO pathway, and inhibition of PDH via *PDK4* expression caused lactate accumulation, as did an up‑regulation of LDH (LDHA). Common associations between metabolic regulators and FAO genes are shown; dark gray indicates genes significantly altered in DEHP samples compared with controls. Numbers indicate steps in the metabolism of FAs, described in “Discussion.” Phthalates can activate nuclear receptors (PPARα, estrogen related receptor gamma; ESRRG) and their coactivators (PGC-1α). PGC-1α and PPARα are associated with increased FAO, mitochondria (mito) biogenesis, and lactate transport. AMP-activated kinase (AMPK) activity can also increase FAO gene and *PDK4* expression via PGC-1α and PPARα. *p < 0.05 compared with the control.

*Increased proton production.* FAO is inversely proportional to glucose oxidation. By-products of FAO inactivate the pyruvate dehydrogenase complex (PDC), thereby diverting pyruvate to lactate via LDH. Therefore, increased FAO can uncouple glycolysis from glucose oxidation, leading to proton and lactate accumulation (Figure 6D). To confirm this pathway, we performed pH measurements on treated cells. After 3 days of treatment, cells exposed to a high concentration of DEHP (100 µg/mL) showed significant extracellular acidosis (Figure 6A). Acidosis increased with time. After 6 days of treatment, cell samples treated with 100 μg/mL DEHP reached a pH of 5, whereas cell samples treated with 50 μg/mL DEHP were also more acidic, although to a lesser extent. However, Wy-14643–treated samples were more basic (pH 7.5) compared with controls. The observed pH changes were not attributed to chemical additives (Figure 6A). Because pH changes can also reflect cell culture overgrowth or cell death, we examined cell viability to rule out these effects. Wy-14643–treated samples had a decrease in cell viability (2.5% of control) and total cell number (20% of control), which may explain the shift to a more basic pH. However, neither cell viability nor total cell number was altered in DEHP-treated samples (Figure 6B,C). DEHP-induced acidosis was independently verified in extracellular flux experiments; DEHP samples were more acidic than controls after 1 hr incubation in unbuffered media (data not shown).

## Discussion

DEHP is a widely used phthalate found in FDA-approved medical devices, tubing, and bags for intravenous fluids. DEHP toxicity has been reported for multiple organs in animals (Carlson 2010), and human studies have raised concerns pertaining to the male reproductive tract and development (Swan 2008). In addition, public health studies have correlated phthalate exposure with metabolic disorders in humans (Stahlhut et al. 2007; Svensson et al. 2011). In contrast, the cardiac effects of DEHP remain largely ignored, and little has been published on the effects of DEHP on cardiac metabolism (Itsuki-Yoneda et al. 2007; Reubsaet et al. 1990). To the best of our knowledge, our study is the first to thoroughly assess the effects of DEHP on substrate metabolism in cardiac cells at clinically relevant doses.

DEHP absorbed by the body is metabolized to mono(2-ethylhexyl)phthalate (MEHP) and 2-ethylhexanol. Oral exposure results in rapid enzymatic hydrolysis to MEHP. However, the rate of DEHP conversion to MEHP is substantially slower after intravenous exposure. In clinical settings DEHP exposure primarily occurs via the intravenous route, which avoids rapid first-pass metabolism (i.e., intravenous fluids, extracorporeal membrane oxygenation, or cardiopulmonary bypass circuits) (Carlson 2010; FDA 2002). Our previous study (Gillum et al. 2009) revealed that DEHP, and not MEHP, adversely affects the electrical properties of cardiac cells (i.e., slowed conduction velocity, induced asynchronous beating, diminished connexin-43 expression). However, DEHP’s metabolites may also influence cardiac metabolism; indeed, MEHP exposure also affected metabolic gene expression [see Supplemental Material, Figure S1 (http://dx.doi.org/10.1289/ehp.1205056)].

We showed that DEHP treatment of cardiomyocytes results in increased expression of genes associated with FA metabolism. Figure 6D illustrates genes of interest for which expression was modified in our experiments (Lopaschuk et al. 2010). Myocyte metabolism of FAs proceeds as follows: FAs enter the myocyte (step 1; Figure 6D) via fatty acid translocase (CD36) and are transported by FA binding protein (FABP). Once inside the cell, FAs undergo esterification (step 2). ACSL1, the predominant isoform found in the heart (Durgan et al. 2006), catalyzes the synthesis of acyl-CoA from FAs; cardiac overexpression of ACSL can result in severe cardiomyopathy due to increased FA uptake and an ensuing accumulation of lipids (Chiu et al. 2001). Acyl-CoA is then converted to acyl-carnitine by CPT1 (step 3), which facilitates entry into the mitochondria. CPT1 activity is tightly controlled by its inhibitor, malonyl-CoA (step 4), which is regulated by AMP-activated kinase (AMPK). AMPK promotes conversion of malonyl-CoA to acetyl-CoA (step 5), thereby alleviating the inhibitory signal on CPT1. Acyl-carnitine is translocated across the mitochondrial inner membrane (step 6) by acylcarnitine translocase (SLC25A20). Once inside the mitochondria, FAs enter the β-oxidation spiral (step 7), whereby acyl-CoA molecules are converted to acetyl-CoA. This conversion is catalyzed by numerous enzymes, including Acadl, Acadvl, Hadha, Hadhb, Decr1, and Acaa2. Each cycle through the β-oxidation spiral produces one molecule each of FADH_2_, NADH, and acetyl-CoA (step 8), and acetyl-CoA is transferred to the citric acid cycle for ATP production. FADH_2_ and NADH by-products are then transferred to the electron transport chain (step 9). Overall, our data show that DEHP treatment influences nearly every step in the FA metabolic pathway.

In DEHP-treated samples, we observed a decrease in oxygen consumption in the presence of glucose, as well as an increase in proton generation. These results confirm that FAO inhibited glucose oxidation, a phenomenon known as the “Randle cycle” (Randle et al. 1963). Increased FAO leads to inhibition of PDC, which is a multienzyme complex that converts pyruvate to acetyl-CoA, thereby linking glycolysis with the citric acid cycle. By-products of FAO increase activity of pyruvate dehydrogenase kinases (PDK), which functions to inactivate the PDC enzyme, pyruvate dehydrogenase (PDH; step 10). Inactivation of PDC prevents the conversion of pyruvate to acetyl-CoA, and instead diverts pyruvate to lactate production via LDH (step 11). Consequently, an increase in FAO and a decrease in PDC activity uncouple glycolysis and glucose oxidation. This scenario, whereby glycolysis is unaltered but pyruvate oxidation is inhibited, can result in an accumulation of protons and lactate. Notably, increased lactate levels have been observed in the muscle of DEHP-treated animals (Martinelli et al. 2006). In the present study, we found that DEHP treatment resulted in an up-regulation in both LDH (microarray analysis; data not shown) and PDK4. This change in gene expression likely explains the extracellular acidosis observed in DEHP samples. A similar phenomenon was observed when adipocytes were exposed to MEHP, which led to increased PDK4 expression and diverted pyruvate away from the citric acid cycle (Ellero-Simatos et al. 2011).

We hypothesize that the described changes in metabolism are partially, but not exclusively, related to up-regulation of *PPAR*α and its coactivator *PGC-1*α. Both PPARα and PGC-1α act as lipid sensors that modify transcriptional responses to metabolic status (Lopaschuk et al. 2010). PPARα is abundant in metabolically active tissues, such as the heart, and we observed DEHP-induced modifications to a number of PPARα target genes (Figure 6D). The influence of PPARα on metabolism has been elucidated in both gain- and loss-of-function experiments. Transgenic mice with cardiac-restrictive *PPAR*α overexpression develop a metabolic phenotype that mimics the diabetic heart, including high FAO rates, low glucose oxidation rates, and gene expression changes including *PDK4* up-regulation (Finck et al. 2002). Moreover, hearts overexpressing PPARα showed signs of cardiomyopathy. Treatment with the PPARα agonist Wy-14643 similarly increased *PDK4* expression in both cardiac (Zungu et al. 2009) and skeletal muscle (reviewed by Burri et al. 2010). In comparison, *PPAR*α-null mice have decreased rates of FAO and increased rates of glucose oxidation (Campbell et al. 2002).

Phthalates are known PPARα agonists (Bility et al. 2004), and many of the effects observed in our studies can be attributed to PPARα up-regulation. However, DEHP-induced toxicity has also been reported in multiple organs of *PPAR*α-null mice (Ito et al. 2007; Peters et al. 1997; Ward et al. 1998). The genes modified by DEHP in *PPAR*α-null mice included those involved in xenobiotic metabolism, including targets of constitutive androstane receptor (CAR) or pregnane X receptor (PXR), and cholesterol biosynthesis which is regulated by other transcription factors, including retinoid X receptor (RXR) (Ren et al. 2010). This is of interest to our studies, as our data illustrated differences between DEHP and PPARα agonist treatment (Wy-14643), suggesting involvement of an alternative receptor.

We propose that increased AMPK expression is an additional mechanism by which DEHP can exert its effects on metabolism. AMPK is an energy sensor: It turns on ATP-producing catabolic processes and turns off ATP-consuming anabolic processes. AMPK can be activated by increased FA supply, increased AMP:ATP ratio, and ischemia. In turn, AMPK activation leads to an up-regulation of PPARα and CPT1β as a way to dispose of FAs via β-oxidation (Lopaschuk et al. 2010). The downstream effects of AMPK activation, including mitochondrial biogenesis, have been attributed to PGC-1α (Jager et al. 2007). Increased AMPK activity was also the main mechanism attributed to an insulin-resistant phenotype observed in PDK4-overexpressing hearts, which included increased FAO, decreased glucose oxidation, and increased lactate levels in myocardial tissue (Chambers et al. 2011). Last, AMPK activity is associated with an inhibition of cholesterol synthesis (Henin et al. 1995), a result we observed in DEHP-treated samples. Despite crosstalk between PPAR and AMPK, PPARα agonists modulate AMPK activity independently of the PPAR nuclear receptor (Chanda et al. 2009).

All-in-all, our data suggest that DEHP exposure results in metabolic remodeling of cardiomyocytes, whereby cardiac cells increase their dependence on FAs for energy production. This dependence on FAs may be regulated at both the gene expression and posttranscription levels. This phenomenon should be examined further in both *in vitro* adult cardiomyocytes and *in vivo*.

## Conclusions

The heart exhibits energy substrate flexibility; however, several conditions are associated with alterations in glucose and/or FA metabolism. Diabetic cardiac dysfunction is partly driven by metabolic abnormalities, chiefly a loss of energy substrate flexibility. The diabetic phenotype consists of a reduced responsiveness to insulin, decreased glucose utilization, and increased dependence on FAs. Chronic dependence on FAs as a fuel source is associated with an accumulation in lipid intermediates, lactate, protons, and reactive oxygen species. Therefore, dependence on FAs can sensitize the heart to ischemic injury and ventricular dysfunction. Diabetic mouse models also display an increase in myocardial oxygen consumption. The metabolic profile seen in DEHP-treated samples has a striking similarity to that seen in diabetic cardiomyopathy (Lopaschuk et al. 2010). It is possible that the adverse metabolic cascade typically initiated by high levels of circulating triglycerides in diabetic patients could also be initiated by chronic phthalate exposure. This parallel to diabetes has important public health implications that should be further addressed.

## Supplemental Material

(61 KB) PDF
