# Supplementary material for: Phthalate Exposure Changes the Metabolic Profile of Cardiac Muscle Cells
Source: Environ Health Perspect. 2012 Jun 6;120(9):1243–51. doi: 10.1289/ehp.1205056 (PMC3440133; doi:10.1289/ehp.1205056)
Supplement: (61 KB) PDF [file ehp.1205056.s001.pdf]

Supplemental Material

Phthalate Exposure Changes the Metabolic Profile of Cardiac Muscle Cells

Nikki Gillum Posnack, Luther M. Swift, Matthew W. Kay, Norman H. Lee, and Narine Sarvazyan

Supplemental Material, Methods.

Chemicals: Collagenase II was obtained from Worthington (Freehold, NJ). Free fatty acid assay kit was obtained from BioVision (Mountain View, CA). Media, Fetal bovine serum, and porcine trypsin were obtained from Gibco BRL (Grand Island, NY). CytoTox-ONE membrane integrity assay was purchased from Promega (Madison, WI). Ssofast QPCR reagent was obtained from Bio-rad (Hercules, CA). MitoXpress probe was purchased from Axxora (San Diego, CA). PPAR-alpha antibody was purchased from Abcam (San Francisco, CA). MitoTracker red and Trizol were purchased from Invitrogen (Eugene, OR). Cy3 donkey anti-rabbit antibody was purchased from Jackson ImmunoResearch (West Grove, PA). Glyceraldehyde-3-Phosphate Dehydrogenase antibody was purchased from Millipore (Billerica, MA). Anti-rabbit IRdye 800CW and anti-mouse IRdye 680LT antibody were purchased from Li-Cor (Lincoln, NE). RIPA buffer and HALT protease/phosphatase inhibitor cocktail were purchased from Pierce (Rockford, IL). XF24 cell culture microplates were a generous gift from Seahorse Bioscience (North Billerica, MA). DEHP (Lot #112K3730), palmitic acid, fatty-acid free bovine serum albumin (BSA) and all other chemicals were obtained from Sigma Chemical (St. Louis, MO) unless specified otherwise.

Supplemental Material, Table S1. Sequences of primers used for qRT-PCR

	Forward primer	Reverse primer
ACAA2	AAGCCGCTAACGAGGCTGGC	AAGCCGCTAACGAGGCTGGC
ACADL	AACATGGCGGCATCGGTGGG	AACATGGCGGCATCGGTGGG
ACSL1	TGAGTGCATAGGCTCGGCGC	TGAGTGCATAGGCTCGGCGC
ACADVL	CCCGAGTGTGGGGCGACAAC	AGTCGCTGGGCAGAGGTAGGC
CPT1β	TCCGAGGCAGGAGCCCCATC	TCCGAGGCAGGAGCCCCATC
ECH1	GAGGCCCTGGACAGTGGGCT	GAGGCCCTGGACAGTGGGCT
HADHA	AACCCAGACCCGGCAGTGCT	AACCCAGACCCGGCAGTGCT
PDK4	GCGCACTTGCTCTCGCTCGA	GCGCACTTGCTCTCGCTCGA
PGC1α	CACCAAGAAAGGGCCGGAGCA	CACCAAGAAAGGGCCGGAGCA
PGC1β	AAGGTCCCACCCCGATCCCG	AAGGTCCCACCCCGATCCCG
PPARα	AGGCCCTGCCTTCCCTGTGA	GCGGGCCACAGAGCACCAAT
Glutamate Dehydrogenase	CTCTGCTGTCCCGCACCCG	GTCGTCTTCGCGGTCGGTGG
18S ribosomal	TAGAGGGACAAGTGGCGTTC	CGCTGAGCCAGTCAGTGTAG

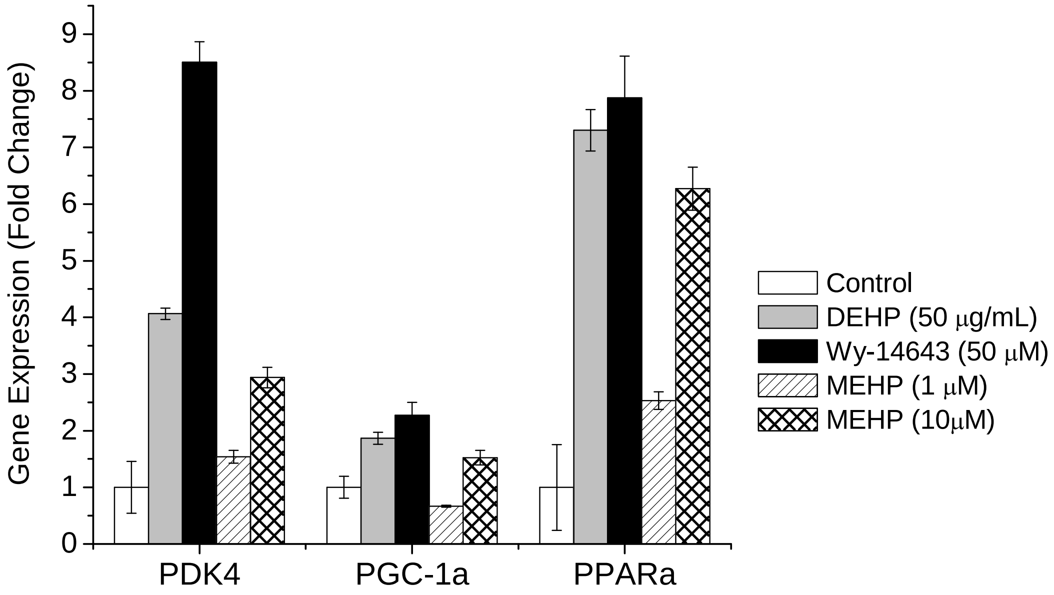

Supplemental Material, Figure S1. qRT-PCR analysis was utilized to compare the expression of PDK4, PGC-1α and PPARα expression in cells treated with DEHP (50 μg/mL), MEHP (1, 10 μM) or the PPARα agonist, Wy-14643 (50 μM). MEHP, a DEHP metabolite, may be responsible for some of the effects observed following DEHP exposure. The expression of fatty acid metabolic genes (PDK4, PGC-1α, PPARα) were upregulated in a dose-response manner following MEHP treatment.
